# Aminoterminal Pro B-Type Natriuretic Peptide (NT-proBNP) Levels for Monitoring Interventions in Paediatric Cardiac Patients with Stenotic Lesions

**DOI:** 10.1155/2009/241376

**Published:** 2010-02-24

**Authors:** Eva Welisch, Knut Kleesiek, Nikolaus Haas, Kambiz Norozi, Ralf Rauch, Guido Filler

**Affiliations:** ^1^Department of Paediatric Cardiology, Ruhr University Bochum, North Rhine Westphalia, 32545, Germany; ^2^Department of Paediatrics, Children's Hospital, London Health Science Centre, University of Western Ontario, 800 Commissioners Road East London, ON, Canada N6A 5W9; ^3^Department of Laboratory Medicine, Heart and Diabetes Centre Bad Oeynhausen, Ruhr University Bochum, North Rhine Westphalia, 32545, Germany; ^4^Department of Paediatric Cardiology, Medical School of Hannover, Germany

## Abstract

*Background.* Serum concentration of NT-proBNP correlates well with the severity of cardiac disease in adults. Few studies have been performed on the applicability of NT-proBNP for monitoring children with congenital heart disease. *Objective.* To assess the potential of NT-proBNP for monitoring the success of interventions in children with stenotic cardiac lesions. *Methods.* NT-proBNP was measured in 42 children aged 1 day to 17 years (y) before and 6 to 12 weeks after surgical or interventional correction of obstructive lesions of the heart. Comparison is made with the clinical status and echocardiographic data of the child. *Results.* NT-proBNP levels (median 280, range 10–263,000 pg/mL) were above the reference value in all but 6 patients (pts) prior to the intervention. Higher levels were found in more compromised patients. The 35 children with clinical improvement after the procedure showed a decline of their NT-proBNP level in all but 4 patients, whose levels remained unchanged. Five patients with unchanged gradients despite a therapeutic intervention also demonstrated unchanged NT-proBNP levels after the intervention. Thus, the success rate of the procedure correlated well to clinical and echocardiographic findings. *Conclusion.* NT-proBNP can be used to assess the efficiency of an intervention.

## 1. Introduction

Natriuretic hormones are released by the myocardium secondary to an increased wall tension caused by volume or pressure in order to protect the cardiovascular system. They are well-established markers for diagnosis, prognosis, and risk stratification in adult patients with congestive heart failure. They are easy to measure, provide a useful tool for monitoring therapeutic success, and are evolving as an important prognostic parameter [1–7]. Their release is triggered by norepinephrine, endothelin 1, vasopressin, and arginine. BNP is formed after enzymatic cleavage of pre-proBNP in the active form of BNP and the inactive aminoterminal fragment NT-proBNP. BNP causes vasodilation, natriuresis, and diuresis by inhibiting the release of renin. The dilatation of the vessels is caused by reduced formation of angiotensin II, whereas the diuretic effect is related to a decreased formation of aldosterone. NT-proBNP also modulates the activity of the sympathicoadrenal system by inhibition of the release of endothelin and is known to have antimitogenic properties [8, 9].

While NT-proBNP closely correlates with BNP levels, NT-proBNP exhibits greater stability and thus may serve as a preferred marker to assess cardiac stress. The potential advantages of NT-proBNP, especially for paediatric patients, have been described in detail elsewhere [8]. 

The objectives of the study were to determine the following

1. if there was an association between baseline NT-proBNP levels and clinical status,
2. if there was an association between changes in NT-proBNP level and the hemodynamic and/or clinical response to the intervention.

## 2. Patients and Methods

### 2.1. Patients

The study was approved by the institutional review board of the Heart Centre in Bad Oeynhausen, Germany. Following informed consent, 42 consecutive patients between 1 day and 17 years (median 6 years of age) were prospectively enrolled into the study between February 2007 and June 2008. All patients who presented during the study period with either a left- or right-sided obstructive cardiac lesion that required therapeutic intervention through cardiac catheterization or surgical correction were included. Patients with elevated serum creatinine at presentation were excluded to avoid elevated levels of low molecular weight proteins as a confounding factor. Left-sided obstruction was seen in 25 patients and right-sided obstruction was diagnosed in 17 patients. Nine patients required surgical correction and 33 patients underwent interventional dilatation or stent implantation via therapeutic cardiac catheterization. Patients' characteristics are provided in Tables 1, 2, and 4.

The infants included in the study typically required an urgent therapeutic intervention, whereas older children were scheduled electively. All indications were based on clinical (arterial hypertension, pulselessness, failure to thrive, cardiac and consecutive respiratory failure) and echocardiographic criteria. We used the modified Ross score as introduced by Laeer et al to assess patients up to14 years of age for signs of heart failure (32 pts) [10–12]; see Table 5. A score of 4 and more was considered abnormal. Patients older than 14 years were classified according to the New York Heart Association functional class (10 pts). We defined haemodynamic improvement after a procedure as diminution of a gradient more than 15 mm Hg and/or the improvement of an abnormal Ross score of more than 2 score points.

### 2.2. Echocardiogram

A complete echocardiographic study was performed prior to the procedure and 6 to 12 weeks later.

### 2.3. Blood Sampling and Assays

A peripheral venous blood sample was collected one day prior to the catheterization or surgical intervention and six to twelve weeks after the procedure during an outpatient visit. After centrifugation, plasma samples were stored either at 8°C for less than three days or at −20° for no more than six months, when NT-proBNP was measured by immunoassay in batch. The IMMULITE 2000 System (Diagnostic Products Corporation DPC, Los Angeles, California) was used to measure NT-proBNP in 50 microlitres of heparinized plasma. The imprecision of the assay was 2.8% (range 0.80% to 11%) in the low range (53 pg/mL, *n* = 169) and −2.75% (range 0.32% to −6.22%) in the high range (350 pg/mL, *n* = 169). 

Paediatric reference intervals have previously been published. There is considerable ontogeny of the reference intervals, largely owing to maturation of renal function and to the peripartum circulatory changes. In brief, values of <3.000 pg/mL for newborns, <200 pg/mL for infants aged 28 days to 1 year, 100 pg/mL for children aged 1–10 years and <50 pg/mL for adolescents and adults were considered normal [13–17].

## 3. Statistical Analysis

Patient data were expressed in median and range and when appropriate as mean +/-SD for descriptive statistics. The nonparametric Wilcoxon's matched pairs test was used to compare the levels before and after intervention and Spearman was used to provide the correlation. For all parameters, a value of *P* < .05 was considered statistically significant. The statistical package GraphPad Prism software version 5.01 for Windows (GraphPad Software, San Diego, CA, USA) was used for the statistical analysis.

## 4. Results

**Figure 1:**
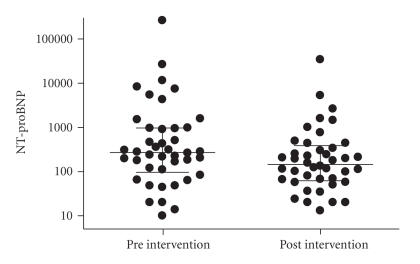
The evolution of the actual NT-proBNP values (log 10, pg/mL) before and after intervention to treat a cardiac stenosis. The difference between the values before and after intervention was highly statistically significant using Wilcoxon's matched pairs test (*P* < .0001).

**Figure 2:**
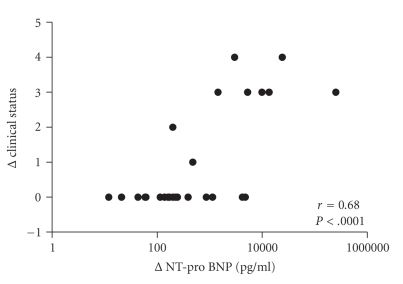
Correlation between change in clinical status and NT-proBNP (log 10, pg/mL).

**Figure 3:**
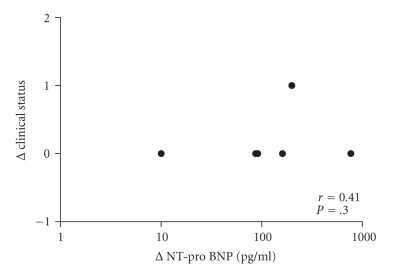
Clinical status and NT-proBNP (log 10, pg/mL) in patients without change after the intervention.

Median concentration of NT-proBNP of all patients prior to intervention was 312 pg/mL (range 10–263,000 pg/mL). After intervention, the concentration dropped to a median of 156 pg/mL (range 20–10,300 pg/mL. Figure 1). The change in NT-proBNP was more obvious in patients who showed significant haemodynamic improvement (delta *P* > 15 mmHg) after procedures (NT-proBNP 409 versus 145 pg/mL), but this change was not statistically significant. There was a good correlation between NT-proBNP and the change of the clinical condition of the patients (modified Ross, Laeer et al., and NYHA) after the intervention: *P* = .0001 Spearman's *r* = 0.68 (see Figure 2). This correlation could not be found in the patients (*n* = 6) who had no significant haemodynamic changes after their procedure (*P* = .3 Spearman's *r* = 0.41, see Figure 3). 

Although some children with left ventricular dysfunction had severely increased NT-proBNP concentrations, the median value for NT-proBNP for left-sided lesions did not differ from right-sided obstruction (*P* = .5053, median of patients with right-sided lesions 193 pg/mL [*n* = 17], median of patients with left-sided lesions 117 pg/mL [*n* = 25]). The characteristics of pts with left- and right-sided obstruction are provided in Table 4.

The improvement of the gradient of patients with a right-sided cardiac stenosis was not as profound as in patients with left-sided stenosis. The movements of the NT-proBNP concentrations were summarized in Tables 2 and 3.

Six patients with isolated coarctation required intervention for arterial hypertension; otherwise they were asymptomatic. Their NT-proBNP was normal at enrolment and remained unchanged after the procedure. 

The patient with a worsening condition demonstrated a parallel rise of NT-proBNP. This patient developed severe pulmonary insufficiency after a surgical procedure, relieving the pulmonary stenosis with a transanular patch. The NT-proBNP level of this patient raised further to 23.100 pg/mL before he subsequently died.

## 5. Discussion

NT-proBNP is well established in adults for clinical management of heart failure. It is a promising marker for monitoring cardiac impairment in children. This has been shown for paediatric patients by Law et al. amongst others [5]. They measured natriuretic hormone levels in pts with known cardiac disease and could demonstrate that the patients with ventricular dysfunction had significantly higher levels. Price and his group demonstrated that the natriuretic hormones were a good predictor of adverse cardiovascular events in paediatric outpatients with left ventricular dysfunction [3].

Our study provides new insight on the impact of a successful intervention for cardiovascular malformations on NT-proBNP levels several months after a procedure. The data suggest that NT-proBNP could be an additional tool for the indication of an intervention as well as a marker to follow patients longitudinally. 

Several studies have been published that examined short-term changes of NT-proBNP after a procedure. They confirmed the perioperative prognostic value of NT-proBNP. Gessler and his colleagues could demonstrate that a high preoperative NT-proBNP level was associated with a complicated postoperative outcome [18]. Walsh and his group found in their study that the NT-proBNP level measured preoperatively in 38 children was a significant predictor for the length of stay on the intensive care unit after surgery and that the peak postoperative level was a significant predictor of the intensity of the overall medical management [19]. Shih et al. supported the value of NT-proBNP as a postoperative marker [20]. They examined 51 patients after congenital heart surgery and found an association between duration of ventilation and a NT-proBNP level > 540 pg/mL with very good sensitivity and specifity. Koch et al. demonstrated in their study that the natriuretic hormones initially increase after cardiac surgery despite haemodynamic unloading and speculated that this could be explained by a cytoprotective role of the natriuretic hormones after surgery [21].

We used reference values that were composed from a variety of articles [13–17] that published previously collected NT-proBNP data in healthy children. It appears that widely accepted age-dependent reference values have yet to be established, but many authors based their clinical studies on the reference data we used. It is reassuring that we found high and clearly abnormal values only in very sick children with significant pathology and abnormal modified Ross Scores. Sequential values appear to be of greater value than a single measurement. This was also shown for cardiac transplant patients—the change of NT-proBNP over time correlated with their wellbeing, not the absolute value [22, 23]. The baseline value may be of prognostic significance. Patients with right-sided residual lesions may often be clinically well. An elevation of NT-proBNP unexplained by echocardiographic findings may point to the need for an additional diagnostic workup, including MRI and/or cardiac catheterization. In particular, peripheral pulmonary artery stenosis with or without unequal perfusion of both lungs can be missed by an echocardiographic study and can only be assessed accurately by other diagnostic means. Khositseth et al. support these findings in their study. They examined twenty-one patients after Fallot repair with pulmonary regurgitation. They reported that an NT-proBNP cutoff value of 115 pg/mL can be used as a marker for detection of right ventricular dilation and dysfunction [24].

The general picture that a successful intervention correlates with a substantial reduction of NT-proBNP values did not apply to a few adolescents with isolated coarctation who had a normal NT-proBNP to start with. Their indication for an intervention was related to arterial hypertension rather than any element of cardiac compromise. Studies in adults with aortic stenosis made similar observations. In patients with preserved function of the left ventricle despite a significant pressure gradient, NT-proBNP was in a normal range. The authors postulated that therapeutic intervention might only be indicated in adult patients with aortic stenosis with elevated NT-proBNP levels [1, 2]. We conclude that elevated NT-proBNP measurements in patients with coarctation implied more severe disease with signs of compromised systemic perfusion and failing left ventricle as these patients had an elevated modified Ross Score.

There is currently some controversy whether obesity may influence NT-proBNP levels although one recent study has called this into question [25]. Of note, none of our patients had a BMI above the 95th percentile for age (data not shown). 

### 5.1. Limitations

Despite these promising findings, our study has a few limitations. Kidney function was not specifically determined, but all patients had normal serum creatinine values (data not shown). Despite the possible accumulation of small molecular weight proteins in chronic kidney disease, newer data in adults suggest that NT-proBNP is helpful in determining significant cardiac illness in adults unless renal function is severely impaired [26, 27]. 

The entire study group is small and not very homogeneous in terms of age, disease severity, type of lesion, and type of intervention.

The patients with left-sided lesions seemed to have similar NT-proBNP levels at the first glance compared to the patients with the right-sided lesions, but the patient group with left-sided obstruction has been very inhomogeneous as has been shown in Table 4.

## 6. Conclusion

NT-proBNP is a valuable additional tool to assess the severity of a cardiac illness in children. A decline in NT-proBNP appears to be a marker for successful intervention. Similarly, a normal NT-proBNP may suggest room to delay an intervention in the absence of clinical symptoms.

## 7. Summary

In adults, NT-proBNP is an important biomarker for monitoring the severity of cardiac disease and its therapeutic response. In children, data remain limited due to smaller numbers and inhomogeneous groups of patients. We therefore studied NT-proBNP in 42 children with stenotic cardiac lesions before and after therapeutic intervention. NT-proBNP values were correlated with the clinical presentation and echocardiographic data. NT-proBNP correlated well with the success of the procedure and may help with the monitoring of therapeutic interventions.

## Figures and Tables

**Table 1:** Underlying cardiac diagnosis of investigated patients.

*Left-sided obstruction*	29

Coarctation	19
Aortic stenosis	4
Subaortic stenosis	2

*Right-sided obstruction*	17

Fallot/Pulmonary Atresia after repair with pulmonary artery stenosis or conduit stenosis	10
Pulmonary valve stenosis	4
d-Transposition of the Great Arteries after Switch Repair with pulmonary artery stenosis	2
Midventricular stenosis after ventricular septal defect repair	1

**Table 2:** Patient characteristics, clinical and echocardiographic data in median with range.

Variable (unit)	All patients (*n* = 42)	Left stenosis (*n* = 25)	Right stenosis (*n* = 17)	Symptomatic* (*n* = 27)	Asymptomatic (*n* = 15)
Median age: years (range)	4.8 (0–17)	3 (0–16)	6.5 (0–17)	4.4 (0–16)	11.3 (0–17)

Surgical versus catheterization interventions:	9/33	7/18	2/15	8/19	1 /14

Median peak gradient before intervention (mm Hg per catheterization and/or ECHO in pts scheduled for operation)	64 (10–120)	50 (20–125)	85 (10–120)	64 (20–120)	64 (10–125)

Median peak gradient after intervention (mm Hg per catheterization and/or ECHO in patients scheduled for operation)	10 (0–90)	0 (0–40)	36 (0–90)	10 (0–80)	20 (0–90)

Median NT-proBNP before (pg/mL)	280 (10–263000)	263 (10–263000)	312 (48–978)	1570 (120–263.000)	125 (10–944)

Median NT-proBNP after (pg/mL)	134 (10–34300)	117 (20–34300)	193 (69–490)	480 (20–34300)	115 (20–490)

*Symptomatic: increased modified Ross Score in pts 0–14 y or NYHA in pts > 14 y or arterial hypertension and diminished pulses in patients with coarctation.

**Table 3:** Change of NT-proBNP concentrations (pg/mL) before and after intervention.

Hemodynamic status	Number of patients	NT-proBNP (pg/mL) (median and range)			Peak gradient (mm Hg) (median and range)		
		Before	After	*P*	Before	After	*P*
Unchanged	6	209 (10–460)	202 (20–338)	ns	40 (10–100)	33 (10–90)	ns
Improvement	35	409 (20–263.000)	145 (20–10.300)	<.0001	64 (20–125)	10 (0–90)	<.0001

Unchanged indicates patients with dP <15 mmHg after procedure.

**Table 4:** Comparison of patients with left-sided obstruction and right-sided obstruction.

Site of obstruction	Number of patients	NT-proBNP (pg/mL)
Left Side	Total: 25	
	Neonates: 3	5437,11487 and 263.000
	1–12 months: 7	204–13.779, median1570
	1–10 years: 10	48–26430, median 108
	Adolescents: 5	10–147, median 20

Right Side	Total: 17	
	Neonates: 2	978 and 4290
	1–12 months: 2	239 and 358
	1–10 years: 6	224–944, median 445
	Adolescents: 7	48–897, median 183

**Table 5:** Clinical score modified from Ross and Reithmann et al. by Laeer et al.

		Score (points)	
	0	1	2
History:			
Diaphoresis	Head only	Head and body during exercise	Head and body at rest

Tachypnea	Rare	Several times	Frequent

Physical examination:			
Breathing	normal	Retractions	Dyspnea

Respiratory rate (respirations/min)			
0–1 y	<50	50–60	>60
1–6 y	<35	35–45	>45
7–10 y	<25	25–35	>35
11–14 y	<18	18–28	>28

Heart rate (beats/min)			
0–1 y	<160	160–170	>170
1–6 y	<105	105–115	>115
7–10 y	<90	90–100	>100
11–14 y	<80	80–90	>90

Hepatomegaly			
(liver edge from right costal margin)	<2 cm	2–3 cm	>3 cm

## References

[1] Bergler-Klein J, Klaar U, Heger M (2004). Natriuretic peptides predict symptom-free survival and postoperative outcome in severe aortic stenosis. *Circulation*.

[2] Gerber IL, Stewart RAH, Legget ME (2003). Increased plasma natriuretic peptide levels reflect symptom onset in aortic stenosis. *Circulation*.

[3] Price JF, Thomas AK, Grenier M (2006). B-type natriuretic peptide predicts adverse cardiovascular events in pediatric outpatients with chronic left ventricular systolic dysfunction. *Circulation*.

[4] Law YM, Ettedgui J, Beerman L, Maisel A, Tofovic S (2006). Comparison of plasma B-type natriuretic peptide levels in single ventricle patients with systemic ventricle heart failure versus isolated cavopulmonary failure. *American Journal of Cardiology*.

[5] Law YM, Keller BB, Feingold BM, Boyle GJ (2005). Usefulness of plasma B-type natriuretic peptide to identify ventricular dysfunction in pediatric and adult patients with congenital heart disease. *American Journal of Cardiology*.

[6] Holstroem H, Hall C, Thaulow E (2001). Plasma levels of natriuretic peptides and hemodynamic assessment of patent ductus arteriosus in preterm infants. *Acta Paediatrica*.

[7] Koch A, Zink S, Singer H (2006). B-type natriuretic peptide in paediatric patients with congenital heart disease. *European Heart Journal*.

[8] Daniels LB, Maisel AS (2007). Natriuretic peptides. *Journal of the American College of Cardiology*.

[9] Costello JM, Goodman DM, Green TP (2006). A review of the natriuretic hormone system’s diagnostic and therapeutic potential in critically ill children. *Pediatric Critical Care Medicine*.

[10] Ross RD, Bollinger RO, Pinsky WW (1992). Grading the severity of congestive heart failure in infants. *Pediatric Cardiology*.

[11] Laeer S, Mir TS, Behn F (2002). Carvedilol therapy in pediatric patients with congestive heart failure: a study investigating clinical and pharmacokinetic parameters. *American Heart Journal*.

[12] Reithmann C, Reber D, Kozlik-Feldmann R (1997). A post-receptor defect of adenylyl cyclase in severely failing myocardium from children with congenital heart disease. *European Journal of Pharmacology*.

[13] Johns MC, Stephenson C (2008). Amino-terminal pro-B-type natriuretic peptide testing in neonatal and pediatric patients. *American Journal of Cardiology*.

[14] Mir TS, Flato M, Falkenberg J (2006). Plasma concentrations of N-terminal brain natriuretic peptide in healthy children, adolescents, and young adults: effect of age and gender. *Pediatric Cardiology*.

[15] Mir TS, Marohn S, Laeer S, Eiselt M, Grollmus O, Weil J (2002). Plasma concentrations of N-terminal pro-brain natriuretic peptide in control children from the neonatal to adolescent period and in children with congestive heart failure. *Pediatrics*.

[16] Nir A, Bar-Oz B, Perles Z, Brooks R, Korach A, Rein AJJT (2004). N-terminal pro-B-type natriuretic peptide: reference plasma levels from birth to adolescence. Elevated levels at birth and in infants and children with heart diseases. *Acta Paediatrica*.

[17] Soldin SJ, Soldin OP, Boyajian AJ, Taskier MS (2006). Pediatric brain natriuretic peptide and N-terminal pro-brain natriuretic peptide reference intervals. *Clinica Chimica Acta*.

[18] Gessler P, Knirsch W, Schmitt B, Rousson V, von Eckardstein A (2006). Prognostic value of plasma N-terminal pro-brain natriuretic peptide in children with congenital heart defects and open-heart surgery. *Journal of Pediatrics*.

[19] Walsh R, Boyer C, LaCorte J (2008). N-terminal B-type natriuretic peptide levels in pediatric patients with congestive heart failure undergoing cardiac surgery. *Journal of Thoracic and Cardiovascular Surgery*.

[20] Shih C-Y, Sapru A, Oishi P (2006). Alterations in plasma B-type natriuretic peptide levels after repair of congenital heart defects: a potential perioperative marker. *Journal of Thoracic and Cardiovascular Surgery*.

[21] Koch A, Kitzsteiner T, Zink S, Cesnjevar R, Singer H (2007). Impact of cardiac surgery on plasma levels of B-type natriuretic peptide in children with congenital heart disease. *International Journal of Cardiology*.

[22] Lindblade CL, Chun DS, Darragh RK, Caldwell RL, Murphy DJ, Schamberger MS (2005). Value of plasma B-type natriuretic peptide as a marker for rejection in pediatric heart transplant recipients. *American Journal of Cardiology*.

[23] Lan Y-T, Chang R-KR, Alejos JC, Burch C, Wetzel GT (2004). B-type natriuretic peptide in children after cardiac transplantation. *Journal of Heart and Lung Transplantation*.

[24] Khositseth A, Manop J, Khowsathit P (2007). N-terminal pro-brain natriuretic peptide as a marker in follow-up patients with tetralogy of fallot after total correction. *Pediatric Cardiology*.

[25] Cortes R, Otero MR, Morillas P (2008). Obese and nonobese patients with essential hypertension show similar N-terminal proBNP plasma levels. *American Journal of Hypertension*.

[26] Bruch C, Fischer C, Sindermann J, Stypmann J, Breithardt G, Gradaus R (2008). Comparison of the prognostic usefulness of N-terminal pro-Brain natriuretic peptide in patients with heart failure with versus without chronic kidney disease. *American Journal of Cardiology*.

[27] Das SR, Abdullah SM, Leonard D (2008). Association between renal function and circulating levels of natriuretic peptides (from the Dallas heart study). *American Journal of Cardiology*.

